# Comparison of V̇O_2_-Kinetic Parameters for the Management of Heart Failure

**DOI:** 10.3389/fphys.2021.775601

**Published:** 2021-11-25

**Authors:** Jonathan Wagner, Max Niemeyer, Denis Infanger, Otmar Pfister, Jonathan Myers, Arno Schmidt-Trucksäss, Raphael Knaier

**Affiliations:** ^1^Division of Sports and Exercise Medicine, Department of Sport, Exercise and Health, University of Basel, Basel, Switzerland; ^2^Department of Medicine, Training and Health, Institute of Sports Science and Motologie, Philipps-University Marburg, Marburg, Germany; ^3^Department of Cardiology, University Hospital Basel, University of Basel, Basel, Switzerland; ^4^Cardiology Division, Veterans Affairs (VA) Palo Alto Health Care System, Stanford University, Palo Alto, CA, United States

**Keywords:** V̇O_2max_, V̇O_2_-kinetics, CRF, risk stratification, heart failure

## Abstract

**Objective:** The aim of this study was to analyze whether V̇O_2_-kinetics during cardiopulmonary exercise testing (CPET) is a useful marker for the diagnosis of heart failure (HF) and to determine which V̇O_2_-kinetic parameter distinguishes healthy participants and patients with HF.

**Methods:** A total of 526 healthy participants and 79 patients with HF between 20 and 90 years of age performed a CPET. The CPET was preceded by a 3-min low-intensity warm-up and followed by a 3-min recovery bout. V̇O_2_-kinetics was calculated from the rest to exercise transition of the warm-up bout (on-kinetics), from the exercise to recovery transition following ramp test termination (off-kinetics) and from the initial delay of V̇O_2_ during the warm-up to ramp test transition (ramp-kinetics).

**Results:** V̇O_2_ off-kinetics showed the highest *z*-score differences between healthy participants and patients with HF. Furthermore, off-kinetics was strongly associated with V̇O_2peak_. In contrast, ramp-kinetics and on-kinetics showed only minimal *z*-score differences between healthy participants and patients with HF. The best on- and off-kinetic parameters significantly improved a model to predict the disease severity. However, there was no relevant additional value of V̇O_2_-kinetics when V̇O_2peak_ was part of the model.

**Conclusion:** V̇O_2_ off-kinetics appears to be superior for distinguishing patients with HF and healthy participants compared with V̇O_2_ on-kinetics and ramp-kinetics. If V̇O_2peak_ cannot be determined, V̇O_2_ off-kinetics provides an acceptable substitute. However, the additional value beyond that of V̇O_2peak_ cannot be provided by V̇O_2_-kinetics.

## Introduction

The incidence and prevalence of heart failure (HF) are high and continue to increase in the developed world with aging of the population. Concomitant deaths and healthcare costs related to this syndrome are increasing (Virani et al., 2020). Accurate diagnostic and risk assessment methods for HF are essential to guide clinical decisions for therapeutic strategies with the ultimate goal of decreasing risk and improving health outcomes. Gas exchange variables obtained through cardiopulmonary exercise testing (CPET) are an established method for accurately stratifying risk in patients with HF; many CPET responses now have a substantial evidence base (Wagner et al., 2018). However, maximal CPET parameters can be difficult to interpret as they are highly dependent on subject effort (Wagner et al., 2020). As many patients with HF are not familiar with severe exercise intensities, pushing these patients to their physiological limit remains a challenge. Therefore, there have been many efforts to investigate submaximal markers such as oxygen uptake kinetics (V̇O_2_-kinetics) in patients with HF.

V̇O_2_-kinetics represent the rate at which generation of aerobic adenosine triphosphate (ATP) adjusts to changes in the exercise intensity (Poole and Jones, 2012). This parameter depends on the ability of the cardiovascular system to rapidly increase or decrease the oxygen supply to the working muscles (Kemps et al., 2009; Chatterjee et al., 2013) as well as on the ability of the muscles to rapidly utilize oxygen (Weiss et al., 2017). Therefore, V̇O_2_-kinetics can provide critical information regarding the regulating capacity of the cardiovascular system and the skeletal muscles to utilize oxygen (Chatterjee et al., 2013) and, thus, exercise intolerance and functional mobility (Sietsema et al., 1994; Pavia et al., 1999; Hummel et al., 2016).

Studies investigating whether V̇O_2_-kinetics is a useful marker for risk stratification in HF have reported conflicting findings (de Groote et al., 1996; Meyer et al., 1998; Pavia et al., 1999; Nanas et al., 2001; Schalcher et al., 2003; Fortin et al., 2015; Hummel et al., 2016). While some have reported that the prognostic value of V̇O_2_-kinetics is even superior to V̇O_2peak_ (Schalcher et al., 2003; Fortin et al., 2015), others have reported only moderate or minimal additional value beyond V̇O_2peak_ (de Groote et al., 1996; Pavia et al., 1999; Hummel et al., 2016). These conflicting results are likely caused by the fact that varying features of V̇O_2_-kinetics were analyzed (i.e., on-kinetics or off-kinetics), and different calculation approaches were used.

V̇O_2_-kinetics is traditionally measured by performing a constant load test. CPET using a ramp protocol is, however, the preferred method to perform an exercise test in the clinical setting (Ross et al., 2016). The current manuscript therefore focused only on the utility of V̇O_2_-kinetics during a ramp protocol. The aims of the study were (1) to analyze whether V̇O_2_-kinetics parameters obtained from a CPET can distinguish between healthy participants and cardiac patients with HF and between New York Heart Association (NYHA) functional classes; (2) to determine which V̇O_2_-kinetic parameter and which calculation are most useful; and (3) whether the most promising V̇O_2_ on- and V̇O_2_ off-kinetic parameter can add additional value to V̇O_2peak_.

## Materials and Methods

### Cohort and Recruitment

The COmPLETE-Study is a cross-sectional single-center study and consists of two parts, namely, COmPLETE-Health and COmPLETE-Heart. COmPLETE-Health included healthy men and women without any known exercise-limiting diseases between 20 and 90 years of age equally distributed across age decades and sex. COmPLETE-Heart included cardiac patients with stable HF with NYHA functional classes I–III, with symptoms and signs stable for at least 1 month. Diagnosis of HF was confirmed by clinical history, physical examination, assessment of natriuretic peptide (NT-proBNP), and echocardiographically documented structural heart disease or diastolic dysfunction according to the European Society of Cardiology guidelines (Ponikowski et al., 2016). Details on recruitment procedures and complete inclusion and exclusion criteria can be found in the study protocol (Wagner et al., 2019).

### Setting

The study was carried out at the Department of Sport, Exercise, and Health at the University of Basel, Switzerland, and was funded by the Swiss National Science Foundation (grant no. 182815). The study complies with the Declaration of Helsinki and was approved by the Ethics Committee of Northwestern and Central Switzerland (EKNZ 2017-01451). Written informed consent was obtained from all participants before the start of the study.

### Acquisition of Participant Characteristics

Resting systolic and diastolic blood pressures were measured with the participant in the supine position using a non-invasive vascular screening system (VaSera VS-1500N; Fukuda Denshi, Tokyo, Japan). Physicians assessed medical history and medications by the questionnaire onsite. Based on clinical data, structured questions, and self-reported exercise tolerance, each patient with HF was assigned to an NYHA functional class by a physician who was blinded to both CPET results and laboratory data. Blood samples were drawn *via* venipuncture by trained medical staff in fasting state (at least 3 h). Samples were immediately centrifuged, the plasma aliquots were frozen at a temperature of −80°C, and all samples were analyzed together after completion of the study.

### Cardiopulmonary Exercise Testing

An exercise test to maximal voluntary exertion using an electromagnetically braked cycle ergometer (Ergoselect 200; Ergoline, Bitz, Germany) was performed according to one of the following five ramp protocols: (i) a 3-min warm-up either unloaded, a load of 10 or 20 W for protocols 1–3, or a load of 50 W for protocols 4 and 5 followed by (ii) a ramp protocol with a linear workload increases of 7, 10, 15, 20, or 30 W/min for protocols 1–5, respectively, followed by (iii) a 3-min recovery phase at the same workload as the warm-up. The protocol was chosen to achieve a duration of approximately 10 min.

Gas exchange and ventilatory variables were analyzed breath-by-breath continuously using a computer-based system (MetaMax 3B; Cortex Biophysik GmbH, Leipzig, Germany). Each test was preceded by a resting period of 3 min to reach steady-state conditions. In the absence of clinical symptoms or electrocardiographic abnormalities, all tests were continued until maximal exertion. Before and during the test, the participants were verbally encouraged to reach maximal exhaustion. Before each test, the equipment was calibrated in standard fashion with reference gas and known volume. V̇O_2peak_ was defined as the highest 30 s average value during the CPET. The slope of ventilation vs. carbon dioxide consumption (V̇E/V̇CO_2_ slope) was calculated from 1 min after beginning of the ramp test up to the respiratory compensation point. As recommended earlier (Gargiulo et al., 2014; Salvioni et al., 2020), we also calculated V̇O_2peak_ and V̇E/V̇CO_2_ slope expressed as percentage of the predicted values. For this purpose, we used our recently published data (Wagner et al., 2021), which are mainly based on the healthy cohort of the present study.

### V̇O_2_-Kinetic Assessment

**FIGURE 1:**
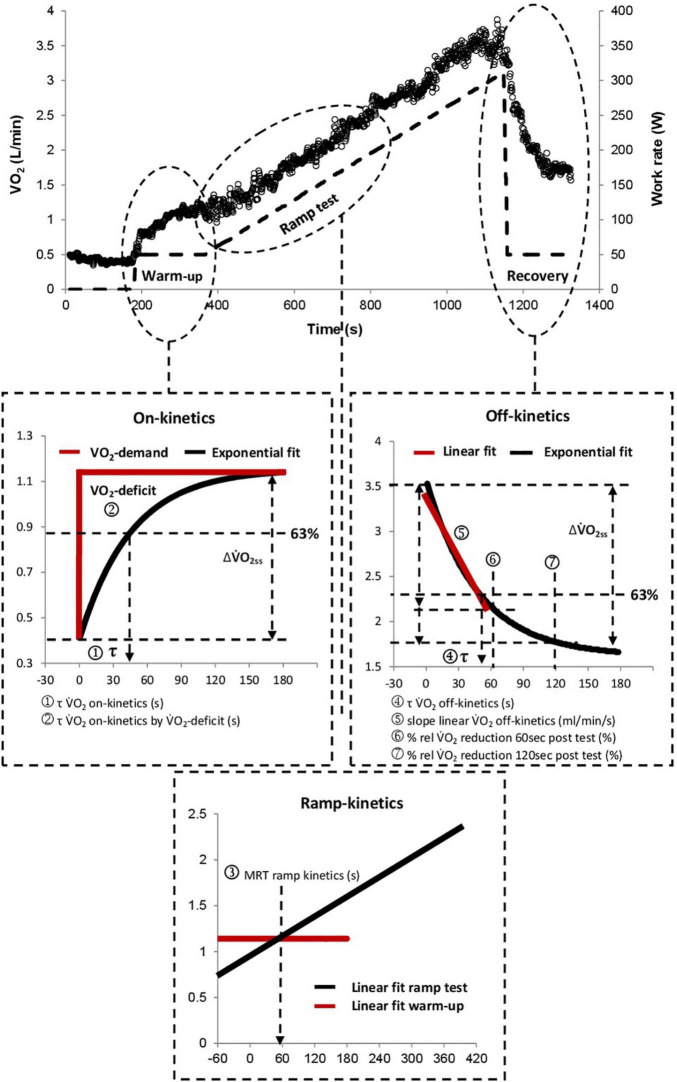
Graphical illustration of all analyzed kinetic parameters. τ *V̇O*_2_ *on-kinetics by V̇O_2_-deficit* was calculated by dividing the V̇O_2_-deficit by the amplitude (ΔV̇O_2ss_) of the V̇O_2_ response. V̇O_2_, oxygen uptake; τ, tau; MRT, mean response time; rel, relative.

Figure 1 displays the different methods used to determine V̇O_2_-kinetics. Initially, V̇O_2_ was filtered by removing all outliers that differed more than three standard deviations from the local mean (moving average of six breaths). The filtered V̇O_2_ values were then linearly interpolated to provide second-by-second values, as previously recommended (Benson et al., 2017). V̇O_2_ on-kinetics was assessed from the rest to exercise transition of the 3-min constant load warm-up period. In accordance with earlier studies, we calculated the time constant of V̇O_2_ on-kinetics by two different approaches:

(1) τ *V̇O*_2_ *on-kinetics*. A mono-exponential function was fit (see Appendix for the exact equation) from the beginning to the end of the warm-up period using non-linear least-squares method regression analyses (Hummel et al., 2016) (see Eq. 1 in Appendix).
(2) τ *V̇O*_2_ *on-kinetics by V̇O*_2_-*deficit*. This was determined by the oxygen deficit and the steady-state increase of V̇O_2_ above the resting value (Sietsema et al., 1994; Schalcher et al., 2003) (see Eq. 2 in Appendix).
  V̇O_2_ off-kinetics was assessed from the active recovery period that directly followed the incremental phase of the CPET. This was done using three different approaches:
(1) τ *V̇O*_2_ *off-kinetics*. Determined by the time constant of a mono-exponential function that was fitted from the beginning to the end of the recovery period using non-linear least-squares method regression analyses (de Groote et al., 1996; Pavia et al., 1999; Hummel et al., 2016) (see Eq. 3 in Appendix).
(2) *Slope linear V̇O*_2_ *off-kinetics*. Determined by the slope of a linear function that was fitted into the V̇O_2_–time relationship of the first minute of recovery using linear least-squares method regression analyses (Nanas et al., 2001) (see Eq. 4 in Appendix).
(3) *% rel V̇O*_2_ *reduction 60 s and 120 s post-test*. Determined by the decrease in V̇O_2_ from the end of the incremental phase up to the first (% rel V̇O_2_ reduction 60 s post-test) and second minute (% rel V̇O_2_ reduction 120 s post-test) expressed as percentages of V̇O_2peak_ (Fortin et al., 2015).

Ramp test kinetics were assessed from the initial delay of V̇O_2_ at the beginning of the incremental exercise phase (mean response time; MRT), as previously described (Meyer et al., 1998; Niemeyer et al., 2020). For this purpose, the intersection between a horizontal line crossing the V̇O_2_ of the final 30 s of the warm-up phase (V̇O_2_ warm-up) and a straight line, which was fitted into the linear V̇O_2_-work rate response of the incremental phase was calculated (see Eq. 5 in Appendix).

### Statistical Analysis

We investigated potential differences in V̇O_2_-kinetics variables between healthy participants and patients with HF using linear regression models, which were adjusted for age and sex. In detail, residual diagnostics were used to see whether the model assumptions were satisfied, and some kinetic parameters were subsequently log-transformed.

To investigate the associations between V̇O_2_-kinetic parameters and V̇O_2peak_, linear regression analyses with V̇O_2peak_ as the dependent variable and age, sex, and the kinetic variables as independent variables were calculated. Separate models for each kinetic parameter were built. Therefore, we modeled age using restricted cubic splines (natural splines) with four knots included along with an interaction by sex to control for potential non-linear age progression (Harrell, 2015). For some models, the residuals exhibited heteroscedasticity, and we present robust *p*-values and confidence intervals for those models (HC3) (Long and Ervin, 2000).

Descriptive statistics were used to compare the V̇O_2_-kinetic variables between NYHA classes I, II, and III and the healthy participants. To achieve comparability, we first created a matched dataset where we matched two healthy participants to every patient with HF according to age and sex (2:1 matching). We used the R package “MatchIt” for these calculations (version 3.0.2) (Ho et al., 2011).

The age- and sex-specific quantile curves were calculated using healthy participants only and applying generalized additive models for location, scale, and shape (GAMLSS, R package version 5.1-6) (Stasinopoulos et al., 2017). The age trajectories were modeled using penalized B-splines (P-splines). We adopted the Bayesian information criterion to select the conditional distribution that offered the best compromise between model complexity and goodness-of-fit. The models were inspected using diagnostic residual plots such as worm plots (van Buuren and Fredriks, 2001) and Q–Q plots. The *z*-scores of the patients with HF were calculated based on the established reference curves using the healthy participants.

Proportional odds ordinal logistic regressions were used to analyze whether the kinetic parameters with the largest mean difference in the *z*-scores added additional predictive information for disease severity (NYHA class). We used the unitless adequacy index to quantify the predictive information contained in V̇O_2peak_, age, and sex compared to the full set of predictors including the kinetic parameters (Harrell, 2015). An adequacy index near one indicates that V̇O_2peak_, age, and sex contain nearly all predictive information already, and that the kinetic parameters add little predictive information. We used likelihood ratio tests to assess whether the kinetic parameters improved the model fit. R version 3.6.1 or later (R Foundation for Statistical Computing, Vienna, Austria) was used for all analyses, and *p*-values ≤ 0.05 were considered statistically significant. All tests were two sided.

## Results

### Participant Characteristics

A total of 526 healthy participants and 79 patients with HF (NYHA functional classes I–III) were included in the study. All patients with HF were in stable condition; their etiologies were cardiomyopathy (*n* = 8), coronary artery disease (*n* = 60), pulmonary hypertension (*n* = 1), valvular regurgitation (*n* = 8), and valvular stenosis (*n* = 2). Thirty-five patients with HF had a preserved ejection fraction (≥50%), 15 patients with HF had mid-range ejection fraction (40–49%), and 23 patients with HF had a reduced ejection fraction (<40%) while the data of six patients with HF were missing. Participant characteristics are presented in Table 1.

**TABLE 1:** Descriptive characteristics of the study population separated into healthy participants and patients with heart failure by NYHA functional classes.

	Healthy		Healthy controls*		NYHA I		NYHA II		NYHA III	
Participants, no. (%)	N	526	N	158	N	37	N	28	N	14
Sex (m/f)		275/251		129/29		35/2		20/8		9/5
Age (yr)	526	54 ± 19.6	158	65.9 ± 13.7	37	65.4 ± 13	28	64 ± 14.3	14	72.9 ± 10.7
Height (cm)	526	171.6 ± 9.2	158	173.9 ± 9	37	174.8 ± 6.6	28	172.1 ± 8.3	14	168.4 ± 9.1
Body mass (kg)	526	69.9 ± 11.6	158	74.8 ± 11.8	37	85.9 ± 14.1	28	84.5 ± 16.4	14	78.4 ± 18.4
BMI (kg/m^2^)	526	23.7 ± 2.7	158	24.7 ± 2.8	37	28.1 ± 4.0	28	28.3 ± 4.0	14	27.7 ± 6.6
Resting systolic BP (mmHg)	525	126.9 ± 13.9	158	131.6 ± 12.8	37	128 ± 13.7	28	127.8 ± 21.9	14	130.1 ± 15.1
Resting diastolic BP (mmHg)	525	77.4 ± 9.0	158	81.4 ± 7.8	37	79.4 ± 12.2	28	77.7 ± 14.4	14	75.9 ± 8.4
Left ventricular ejection fraction (%)		n.a.		n.a.	35	46.4 ± 11.5	26	46.3 ± 11.0	12	44.6 ± 15.4
Etiology, ischemic, n (%)		n.a.		n.a.	37	28 (76)	28	21 (75)	14	11 (79)

**Medication, n (%)**										

Anti-hypertensives (%)	526	46 (9)	158	25 (15)	37	36 (97)	28	25 (89)	14	14 (100)
Of which ACE/ARB (%)	526	44 (8)	158	20 (13)	37	32 (86)	28	21 (75)	14	12 (86)
Beta-blockers (%)	526	12 (2)	158	8 (5)	37	28 (76)	28	22 (79)	14	8 (57)
Anti-coagulants (%)	526	20 (4)	158	13 (8)	37	33 (89)	28	24 (86)	14	12 (86)
Statins (%)	526	22 (4)	158	14 (9)	37	31 (84)	28	20 (71)	14	11 (79)
Diuretics (%)	526	18 (3)	158	10 (6)	37	18 (48)	28	14 (50)	14	9 (64)
Anti-diabetics (%)	526	0 (0)	158	0 (0)	37	6 (16)	28	5 (17)	14	3 (21)

**Blood testing**										

HbA1c (mg/dL)	518	5.2 ± 0.4	157	5.4 ± 0.4	37	5.9 ± 0.7	27	6.0 ± 0.6	13	6.3 ± 0.7
LDL cholesterol (mg/dL)	518	122.6 ± 28.3	157	127.1 ± 26.1	37	89.6 ± 25.3	27	89.8 ± 18.1	13	94.4 ± 34.8
HDL cholesterol (mg/dL)	518	65.6 ± 14.9	157	62.8 ± 13.6	37	50.9 ± 9	27	53.3 ± 12.3	13	55.7 ± 11.7
Total cholesterol (mg/dL)	518	220.2 ± 42.3	157	227.2 ± 39.6	37	168.8 ± 41.3	27	172.4 ± 30.5	13	177.7 ± 54.4
Triglyceride (mg/dL)	518	117.4 ± 62.2	157	127.6 ± 54.4	37	140.1 ± 88.3	27	155.8 ± 109.4	13	103.5 ± 30.2
NTproBNP (pg/mL)	518	121.4 ± 209.6	157	108.3 ± 93.8	37	543.3 ± 573	27	580.0 ± 802.2	13	821.1 ± 655.5

**Performance**										

P_max_ (W)	526	203.2 ± 84.0	158	200.3 ± 80.6	37	153.1 ± 44.0	28	116.1 ± 44.7	14	81.7 ± 30
V̇O_2max_ absolute (L*min^–1^)	526	2.4 ± 0.8	158	2.4 ± 0.8	37	2.0 ± 0.5	28	1.7 ± 0.6	14	1.2 ± 0.3
V̇O_2max_ relative (mL*kg^–1^*min^–1^)	526	34.9 ± 10.3	158	32.5 ± 9.7	37	23.9 ± 5.9	28	19.9 ± 5.6	14	16.3 ± 4.6
% predicted V̇O_2max_ relative	526	101.5 ± 18.4	158	100.6 ± 20.1	37	73.9 ± 21.3	28	62.5 ± 17.3	14	60.1 ± 22.9
% predicted VE/VCO_2_ slope	468	101.0 ± 15.9	130	102.0 ± 15.9	37	137.3 ± 25.2	28	144.2 ± 29.2	14	158.7 ± 34.7
RER_*max*_	526	1.17 ± 0.08	158	1.14 ± 0.08	37	1.09 ± 0.08	28	1.06 ± 0.07	14	1.04 ± 0.08
HR_*max*_ (bpm)	507	169.9 ± 21.1	155	161.5 ± 21.8	37	136.6 ± 20.9	28	137.7 ± 26.5	14	127.2 ± 21.8
V̇O_2_ reduction 60 s post-test (L/min)	506	0.74 ± 0.37	154	0.69 ± 0.34	37	0.46 ± 0.25	28	0.42 ± 0.27	14	0.23 ± 0.19
V̇O_2_ reduction 120 s post-test (L/min)	504	1.19 ± 0.50	154	1.15 ± 0.47	37	0.93 ± 0.34	28	0.76 ± 0.41	14	0.48 ± 0.28

*Data are presented as mean ± standard deviation if not stated otherwise.*

*BMI, body mass index; BP, blood pressure; HR, heart rate; P_max_, maximal power; V̇O_2max_, maximal oxygen uptake; VE, volume of expiration; VCO_2_, carbon dioxide output; RER_max_, maximal respiratory exchange ratio; HR_max_, maximal heart rate; BL_max_, maximal blood lactate.*

*Two participants from the healthy cohort were matched to every patient with heart failure according to age and sex (2:1 matching).

### V̇O_2_-Kinetics in Health and Heart Failure

Group differences between healthy participants and patients with HF irrespective of their NYHA class are reported in Table 2. Six out of eight V̇O_2_-kinetic parameters showed evidence for a difference between the groups (*p* ≤ 0.007). The number of participants involved in the analysis of the respective kinetic parameter indicates the susceptibility to minor measurement difficulties during the CPET and the number of outliers due to the determination method which were excluded.

**TABLE 2:** Group differences between healthy participants and patients with heart failure, *Z*-scores and the association with V̇O_2peak_ for all kinetic parameters.

		Group differences* healthy and HF		*Z*-scores			Association with V̇O2peak^†^		
Parameter	N^†^	Mean difference (95%-CI)	*P*-value	Healthy	HF	Mean difference healthy-HF (95% CI)	Coefficient estimate (95% CI)	Partial R^2^	*P*-value
τ V̇O_2_ on-kinetics (s)	529	0.16 (0.04; 0.28)	0.007	0.00	0.48	−0.48 (−0.79; −0.16)	−0.01 (−0.01; −0.002)	0.04	0.005
τ V̇O_2_ on-kinetics by V̇O_2_-deficit (s)	541	0.07 (−0.00; 0.15)	0.055	0.00	0.43	−0.43 (−0.78; −0.09)	−0.01 (−0.01; −0.003)	0.04	0.000
MRT ramp kinetics (s)	514	0.00 (−0.13; 0.12)	0.963	0.01	0.02	0.02 (−0.30; 0.26)	−0.0003 (−0.01; 0.0005)	0.00	0.899
τ V̇O_2_ off-kinetics (s)	558	0.17 (0.06; 0.27)	0.001	0.00	0.74	−0.74 (−1.04; −0.43)	−0.011 (−0.015; −0.008)	0.09	<0.001
Slope linear V̇O_2_ off-kinetics (ml/min/s)	567	4.96 (3.63; 6.30)	0.000	–0.01	0.88	−0.88 (−1.15; −0.62)	−0.46 (−0.51; −0.41)	0.39	<0.001
% rel V̇O_2_ reduction 60 s post-test (%)	582	−5.76 (−7.94; −3.59)	0.000	0.00	–0.89	0.89 (0.59; 1.18)	0.37 (0.28; 0.46)	0.16	<0.001
% rel V̇O_2_ reduction 120 s post-test (%)	579	−3.54 (−5.84; −1.23)	0.003	0.00	–0.64	0.64 (0.29; 0.98)	0.34 (0.25; 0.44)	0.12	<0.001

*HF, heart failure; τ, tau; MRT, mean response time; V̇O_2_, oxygen uptake; rel, relative.*

*Adjusted for age and sex.

*^†^Including data of healthy participants and patients with heart failure.*

**FIGURE 2:**
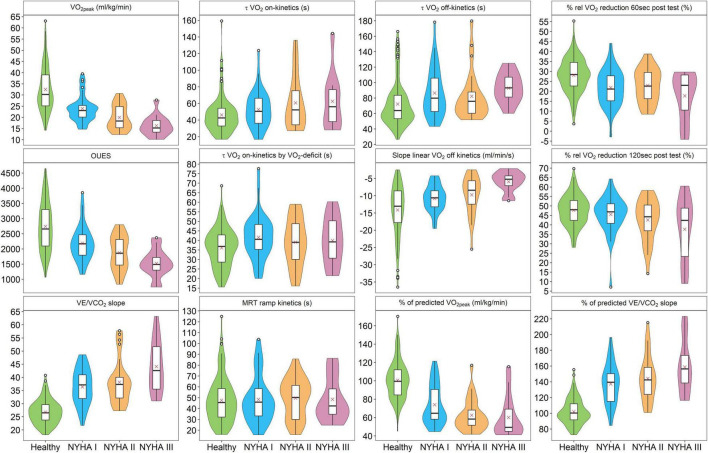
Comparison of VO_2_ on-kinetic und VO_2_ off-kinetic parameters between a healthy control group and patients with heart failure with NYHA functional classes I, II, and III. V̇O_2peak_, peak oxygen uptake; OUES, oxygen uptake efficiency slope; VE, volume of expiration; VCO_2_, carbon dioxide output; τ, tau; MRT, mean response time; rel, relative.

Figure 2 presents violin plots of all analyzed kinetic parameters for NYHA class I, II, and III and the age- and sex-matched healthy reference group. In addition to the kinetic parameters, violin plots were presented for CPET markers known to have high predictive value (Wagner et al., 2018) including V̇O_2peak_, OUES, and V̇E/V̇CO_2_ slope for comparison.

The *z*-scores (Table 2) show that τ *V̇O*_2_ *on-kinetics* was the best V̇O_2_ on-kinetic parameter to discriminate between healthy participants and patients with HF. *Slope linear V̇O*_2_ *off-kinetics (ml/min/s)* and *% rel V̇O*_2_ *reduction 60 s post-test* performed best among the V̇O_2_ off-kinetic parameters. These three parameters were therefore considered superior to the others, and further analyses were limited to these parameters.

**FIGURE 3:**
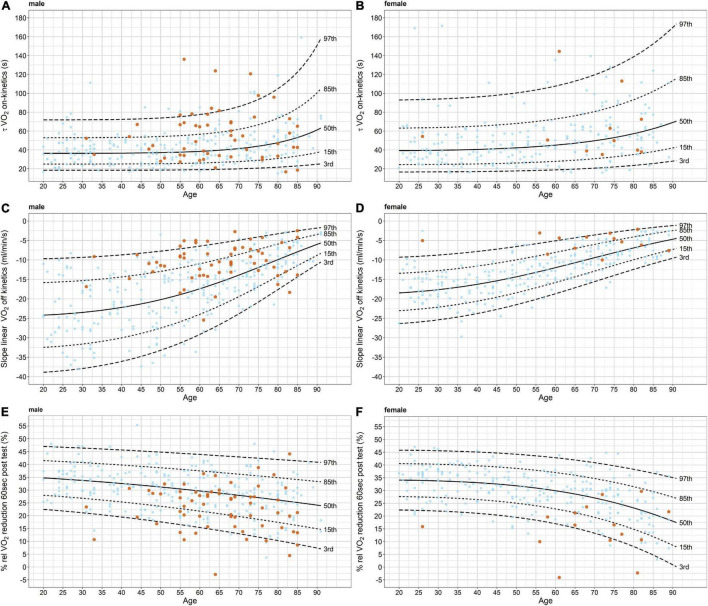
Quantile curves for τ *VO_2_ on-kinetics, slope linear VO_2_ off-kinetics (ml/min/s)*, and *% rel VO_2_ reduction 60 s post-test* for males and females, separately. The quantile curves are based on the healthy participants only (light blue data points). Values of the patients with heart failure are presented in orange. V̇O_2_, oxygen uptake.

Quantile curves for τ *V̇O*_2_ *on-kinetics, slope linear V̇O*_2_ *off-kinetics (ml/min/s)*, and *% rel V̇O*_2_ *reduction 60 s post-test* are presented in Figure 3. The quantile curves based on the healthy participants tend toward pathological numbers with increasing age. For the parameter τ *V̇O*_2_ *on-kinetics*, 60% of the patients with HF were located above the 50th percentile. For the *slope linear V̇O*_2_ *off-kinetics (ml/min/s)*, 85% of the patients with HF were located below the 50th percentile, and for *rel V̇O*_2_ *reduction 60 s post-test*, 78% were located above the 50th percentile.

### Association of V̇O_2_-Kinetics and V̇O_2peak_

There was strong evidence for associations between V̇O_2peak_ (ml/kg/min) and all V̇O_2_ on- and off-kinetics parameter except for *MRT ramp kinetics* in which there was no evidence for such an association observed (Table 2, last three columns). The direction of the association can be described as follows: the faster the V̇O_2_-kinetic response (depending on the parameter, a positive or negative association) the higher the V̇O_2peak_ values are observed. By far, the largest adjusted R^2^ was observed for the *slope linear V̇O*_2_ *off-kinetics (ml/min/s)*.

### Predicting Disease Severity (New York Heart Association-Classification) Using Kinetic Parameters

Table 3 shows the results of several models for NYHA class prediction by kinetic parameters. All kinetic parameters improved the model when the base model contained sex and age. As indicated by the Chi^2^, *slope linear V̇O*_2_ *off-kinetics (ml/min/s)* improved the model from the three kinetic parameters most but not to the extent that V̇O_2peak_ did.

**TABLE 3:** Predicting disease severity (NYHA functional class) using V̇O_2_ kinetic parameters.

Base model	Additional variable	Adequacy of base model	Likelihood ratio test
Sex, age	τ V̇O_2_ on-kinetics (s)	0.88	χ^2^(1) = 7.87, *p* = 0.005
Sex, age	% rel V̇O_2_ reduction 60 s post-test	0.54	χ^2^(1) = 53.81, *p* < 0.001
Sex, age	Slope linear V̇O_2_ off-kinetics (ml/min/s)	0.51	χ^2^(1) = 56.15, *p* < 0.001
Sex, age	V̇O_2peak_	0.27	χ^2^(1) = 176.82, *p* < 0.001

**Additional value**			

V̇O_2peak_, sex, age	τ V̇O_2_ on-kinetics (s)	0.98	χ^2^(1) = 5.36, *p* = 0.02
V̇O2_peak_, sex, age	% rel V̇O_2_ reduction 60 s post-test	0.99	χ^2^(1) = 0.54, *p* = 0.46
V̇O_2peak_, sex, age	Slope linear V̇O_2_ off-kinetics (ml/min/s)	0.99	χ^2^(1) = 0.46, *p* = 0.50

*NYHA class, New York Heart Association-Classification; V̇O_2_, oxygen uptake; τ, tau; rel, relative.*

### Additional Value of Kinetics to Predict Disease Severity

There was little evidence that any of the three kinetic parameters improved the models already containing sex, age, and V̇O_2peak_. The adequacy index comparing the base models containing age, sex, and V̇O_2peak_ to a model additionally containing the kinetic parameters was between 0.98 and 0.99. This means that the base models without the V̇O_2_ kinetic parameters contain nearly all the predictive information already.

## Discussion

To our knowledge, the current study is the first to provide detailed V̇O_2_ kinetic results in large cohorts of healthy participants and patients with HF. All previously suggested methods to calculate V̇O_2_ on- and off-kinetics for risk stratification using a standard ramp protocol were analyzed and compared. Our results show that the V̇O_2_ off-kinetics according to *rel V̇O*_2_ *reduction 60 s post-test (%)* or *slope linear V̇O*_2_ *off-kinetics (ml/min/s)* present an alternative to evaluate aerobic function and disease severity if V̇O_2peak_ cannot be determined. Additional value beyond that of V̇O_2peak_ for risk stratification of patients with HF was not provided by V̇O_2_ on- or off-kinetics.

### Differences Between Healthy Participants and Patients With Heart Failure

This study provides evidence that V̇O_2_-kinetic parameters differ between healthy participants and a group of mild to moderate functionally impaired patients with HF for all kinetic calculation methods with the exception of τ *V̇O*_2_ *on-kinetics by V̇O_2_-deficit* and *MRT of the ramp kinetics*. The observed differences are in line with previous findings showing that patients with HF had significantly slower V̇O_2_-kinetics compared to healthy volunteers (Sietsema et al., 1994; Meyer et al., 1998; Pavia et al., 1999; Hummel et al., 2016). The slowing of V̇O_2_-kinetics in HF is closely related to impaired ventricular-pulmonary vascular function (Kemps et al., 2009; Chatterjee et al., 2013) and/or impaired peripheral oxygen utilization (Weiss et al., 2017).

Mean differences in *z*-scores (Table 2) clearly indicate that V̇O_2_ off-kinetics, irrespective of the calculation method, discriminate better between healthy participants and patients with HF compared to V̇O_2_ on-kinetics.

These results are in line with previous research showing that off-kinetics can be determined with greater fidelity (Kemps et al., 2009) and higher reproducibility (Kemps et al., 2007) than on-kinetics in patients with HF. Further, irrespective of the methodological difficulties with on-kinetics, off-kinetics may discriminate patients with HF better from their healthy counterparts as has been observed in a previous study (Sietsema et al., 1994).

The comparison between the off-kinetics parameters (different calculation approaches) revealed a higher potential to distinguish healthy participants and patients with HF for % *rel V̇O_2_ reduction 60 s post-test* and *slope linear V̇O_2_ off-kinetics* compared to % *rel V̇O_2_ reduction 120 s post-test* and τ *V̇O*_2_ *off-kinetics*. Interestingly, both superior off-kinetics parameters were determined from the first minute of the recovery period only, while the other parameters were calculated from the first 2 min (*% rel V̇O*_2_ *reduction 120 s post-test*) or the entire recovery duration (τ *V̇O*_2_ *off-kinetics*). This indicates that the very early phase of the off transition better distinguished between healthy participants and patients with HF.

### Association With V̇O_2 peak_

Strong significant associations between V̇O_2peak_ and off-kinetics were observed. *Slope linear V̇O*_2_ *off-kinetics* explained 39% of the variation in V̇O_2peak_ among healthy participants and patients with HF. In contrast, V̇O_2_ on-kinetics showed significant but only weak associations with V̇O_2peak_; the on-kinetics parameter τ V̇O_2_ (s) explained only 4% of the variation in V̇O_2peak_. The stronger association of the off-kinetics compared to the on-kinetics can likely be explained by the methodological considerations of the on-kinetics described above.

A recent study showed that the level of exhaustion had no impact on V̇O_2_ off-kinetics (Ichikawa et al., 2020). That the determination of V̇O_2_-kinetics, unlike V̇O_2peak_, does not require the subject to perform the test to maximal voluntary exertion is a large advantage. Many patients lack the motivation to perform a maximal exercise test, are not familiarized with severe exercise, or may have a contraindication to maximal exertion (Green and Askew, 2018). In contrast, the successful determination of V̇O_2peak_ requires either a V̇O_2_-plateau or a confirmation of a secondary exhaustion criteria (Wagner et al., 2020). Considering the large existing evidence base for the valuable information V̇O_2_-kinetics provides coupled with the present results, V̇O_2_ off-kinetics can be suggested as potential substitute for V̇O_2peak_.

### Predicting Disease Severity

The ability of a model to predict health status and disease severity of the patients with HF improved significantly when the V̇O_2_ on-kinetic parameter (τ *V̇O*_2_ *on-kinetics*) and the V̇O_2_ off-kinetic parameter (*% rel V̇O*_2_ *reduction 60 s post-test*) were added. However, only V̇O_2_ off-kinetics added substantial information to the model. Thus, V̇O_2_ off-kinetics could be a tool to discriminate not only between healthy participants and those with mild functional impairment (NYHA class I) but also between NYHA classes as visualized by Figure 2. Our results are in line with previous studies showing the potential of V̇O_2_-kinetics for risk stratification (Schalcher et al., 2003; Fortin et al., 2015) but are in contrast to others who did not demonstrate better predictive value by the addition of V̇O_2_ off-kinetics (de Groote et al., 1996; Pavia et al., 1999; Hummel et al., 2016).

Since we could already show the association with V̇O_2peak_—considered the gold standard criteria for risk stratification—another established parameter, NYHA functional class, was used to stratify risk in patients with HF. Based on the different underlining physiological aspects represented by V̇O_2peak_ and V̇O_2_-kinetics (Sietsema et al., 1994; Chatterjee et al., 2013), some additional predictive value of V̇O_2_-kinetics could be expected. However, our results showed minimal evidence of additional value of V̇O_2_ on- or off-kinetics. Two reasons likely explain these results: (i) V̇O_2peak_ is already a very strong risk predictor in patients with HF and the association of V̇O_2peak_ and NYHA class is already known to be high and (ii) V̇O_2_-kinetics are likely to provide the same predictive information as V̇O_2peak_, which is underscored by the association between V̇O_2_ off-kinetics and V̇O_2*peak*_ in this study. Therefore, even though we observed that V̇O_2_-kinetics has predictive value, it does not appear to have value beyond V̇O_2peak_.

### Practical Applications

Our results indicate that the method of quantifying V̇O_2_-kinetics is critical to its clinical application. They suggest that the determination of V̇O_2_ on-kinetics from rest to a light constant load phase is not optimal; rather, the results favor the analysis of off-kinetics when using a ramp protocol. The calculation of *rel V̇O*_2_ *reduction 60 s post-test (%)* or *slope linear V̇O*_2_ *off-kinetics (ml/min/s)* is recommended to distinguish between healthy individuals and patients with HF. Since V̇O_2_ off-kinetics is not affected by the level of exhaustion (Ichikawa et al., 2020), these parameters might be used as a substitute for V̇O_2peak_ when maximal exhaustion is not reached or when V̇O_2peak_ cannot be interpreted.

Using some basic spreadsheet calculation tools, the calculation of *rel V̇O*_2_ *reduction 60 s post-test (%)* and *slope linear V̇O*_2_ *off-kinetics (ml/min/s)* are quite simple (see Appendix). To facilitate the routine application of V̇O_2_ off-kinetics in the clinical setting, we recommend that the incorporation of these parameters in CPET application software.

## Limitations

Our study has limitations. The study was cross-sectional, and therefore no hard endpoints such as mortality or hospitalization were available. Furthermore, our HF cohort is predominantly represented by male ischemic patients with mildly reduced left ventricular ejection fraction and comparatively preserved exercise capacity as suggested by their mean values of V̇O_2_ peak. This may not fully reflect the real-world HF population, which partly limits the transferability of our findings. To further improve the reliability and validity of the V̇O_2_ on- and off-kinetics determination, a warm-up and a cool-down phase of 5 min instead of 3 min could be applied.

## Conclusion

Differences in V̇O_2_-kinetics between healthy participants and patients with HF are observed and are highly dependent on how they are calculated. V̇O_2_ off-kinetics appears to be superior for distinguishing patients with HF and healthy participants compared with V̇O_2_ on-kinetics and ramp-kinetics. If V̇O_2peak_ cannot be determined, V̇O_2_ off-kinetics provides an acceptable substitute. However, additional value beyond that of V̇O_2peak_ cannot be provided by V̇O_2_-kinetics.

## Data Availability Statement

The raw data supporting the conclusions of this article will be made available by the authors, without undue reservation.

## Ethics Statement

The studies involving human participants were reviewed and approved by the Ethics Committee of Northwestern and Central Switzerland (EKNZ 2017-01451). The patients/participants provided their written informed consent to participate in this study.

## Author Contributions

JW, MN, JM, RK, DI, and AS-T: conception and design of the research and analysis and interpretation of the data. JW and RK: acquisition of data. DI: statistical analysis. AS-T and RK: obtaining funding and supervising the work. JW and MN: drafting the manuscript. DI, JM, OP, AS-T, and RK: critical revision of the manuscript for important intellectual content. All authors contributed to the article and approved the submitted version.

## Conflict of Interest

The authors declare that the research was conducted in the absence of any commercial or financial relationships that could be construed as a potential conflict of interest.

## Publisher’s Note

All claims expressed in this article are solely those of the authors and do not necessarily represent those of their affiliated organizations, or those of the publisher, the editors and the reviewers. Any product that may be evaluated in this article, or claim that may be made by its manufacturer, is not guaranteed or endorsed by the publisher.

## Appendix

### V̇O_2_-Kinetic Calculation

V̇O_2_ on-kinetics:

(1) τ *V̇O*_2_ *on-kinetics*.

(1)
$$\dot{V}O_{2}=\dot{V}O_{2rest}+△\dot{V}O_{2SS}\cdot \left(1-exp^{-\frac{t}{\tau }}\right)$$

where V̇O_2rest_ was defined as the mean of the final 60 s of the resting period preceding the warm-up. ΔV̇O_2SS_ was the steady-state increase of V̇O_2_ above V̇O_2rest_ and τ the time constant of the overall response. τ thereby includes the cardio-dynamic and the primary component of V̇O_2_ on-transient kinetics and is equivalent to that what previously has been also called mean response time (MRT) of square wave exercise.

(2) τ *V̇O*_2_ *on-kinetics by V̇O*_2_-*deficit*.

(2)
$$\tau =\frac{VO_{2}deficit}{△\dot{V}O_{2SS}}$$

where ΔV̇O_2SS_ was previously calculated from the mean V̇O_2_ of the final 30 s of the warm-up period. The V̇O_2_-deficit was calculated from the difference between the consumed V̇O_2_ and the V̇O_2_-demand, which was calculated by multiplying ΔV̇O_2SS_ with the duration of the warm-up period (for further details see: Schalcher et al., 2003).

V̇O_2_ off-kinetics:

(1) τ *V̇O*_2_ *off-kinetics*.

(3)
$$\dot{V}O_{2}=\dot{V}O_{2rec}+△\dot{V}O_{2SS}\cdot \left(exp^{-\frac{t}{\tau }}\right)$$

where V̇O_2rec_ was defined as the asymptotic value of the recovery response. ΔV̇O_2SS_ was the steady-state decrease of V̇O_2_ above V̇O_2*rec*_ and τ the time constant of the overall response.

(2) *Slope linear V̇O*_2_ *off-kinetics*.

(4)
$$\dot{V}O_{2}=at+b$$

where a represents the slope and b the intercept of the linear V̇O_2_–time relationship.

### Mean Response Time Ramp Test

(5)
$$MRTramptest=\frac{\dot{V}O_{2warm-up}-b}{a\;S}-\frac{P_{warm-up}}{S}$$

where V̇O_2 warm–up_ and P_warm–up_ are defined as the V̇O_2_ and the work rate of the warm-up phase preceding the incremental phase. a and b represent the slope and the intercept of the V̇O_2_–work rate relationship of the incremental phase. S is defined as the ramp slope. The V̇O_2_ work rate slope was previously calculated using linear least-squares method regression analyses. To avoid any effects of a non-linear V̇O_2_ response due to the initial lag of V̇O_2_ or a potential plateau, the first minute and the last 2 min were excluded for the calculation.
